# On the Nature of Stationary and Time-Resolved Fluorescence Spectroscopy of Collagen Powder from Bovine Achilles Tendon

**DOI:** 10.3390/ijms24087631

**Published:** 2023-04-21

**Authors:** Łukasz Saletnik, Wojciech Szczęsny, Jakub Szmytkowski, Jacek J. Fisz

**Affiliations:** 1 Faculty of Health Sciences, College of Medicine, Nicolaus Copernicus University, 85-067 Bydgoszcz, Poland; l.saletnik@cm.umk.pl; 2 Faculty of Medicine, College of Medicine, Nicolaus Copernicus University, 85-067 Bydgoszcz, Poland; wojszcz@cm.umk.pl (W.S.); jakub.szmytkowski@cm.umk.pl (J.S.)

**Keywords:** collagen, collagen cross-links, fluorescence stationary spectroscopy, fluorescence time-resolved spectroscopy, fluorescence excitation energy transfer

## Abstract

This paper presents a more systematic study of steady-state and time-resolved autofluorescence spectroscopy of collagen isolated from bovine Achilles tendon. In steady-state fluorescence measurements, the excitation and emission spectra of collagen powder, recorded at different fluorescence excitation and detection wavelengths, were compared with the fluorescence excitation and emission spectra of the amino acids phenylalanine, tyrosine, and tryptophan, as well as with similar spectra for 13 autofluorescent collagen cross-links, which have been identified and described in the literature so far. In time-resolved studies, fluorescence was excited by the pulsed light of different wavelengths, and for each excitation wavelength, fluorescence decay was recorded for several detection wavelengths. Data analysis allowed recovery of the fluorescence decay times for each experimental excitation detection event. The obtained information on the decay times of the measured fluorescent signals was discussed, taking into account the available literature data from similar studies of isolated collagen and collagen-rich tissues. Based on the obtained results, it was found that the shape and position of the measured fluorescence excitation and emission spectra of collagen strongly depend on the emission and excitation wavelengths selected in the measurements. From the recorded excitation and emission bands of collagen, it can be concluded with high probability that there are additional, so far unidentified, collagen cross-links, which can be excited at longer excitation wavelengths. In addition, the collagen excitation spectra were measured at longer emission wavelengths at which the collagen cross-links emit fluorescent light. In addition to the emission spectra obtained for excitation in the deep-UV region, the results of time-resolved fluorescence studies with excitation in the deep-UV region and detection at longer wavelengths suggest that fluorescence excitation energy transfer processes occur from the amino acids to the collagen cross-links, and also between the cross-links themselves.

## 1. Introduction

Collagen is the most widespread protein in the human body. It is the primary ingredient of cartilage, skin, tendons, bones, muscles, and all organs. Changes in this protein may cause many health disorders, from systemic diseases and corneal degeneration to tumors. Collagen is the main component of the extracellular matrix; therefore, during cancer progression and metastasis, quantitative and qualitative changes in collagen occur [1]. Collagen gene mutations and defects in collagen-modifying enzymes can be the causative factor of numerous diseases. Collagen diseases can be autoimmune or hereditary. Autoimmune collagen vascular diseases include lupus, scleroderma, rheumatoid, and temporal arthritis, while hereditary collagen diseases include Marfan’s syndrome and Ehlers–Danlos syndrome. In autoimmune diseases of the connective tissue, the immune system causes inflammation within the collagen. It has been known for several decades that one of the basic causes of hernia formation is disturbances in both the synthesis and degradation of collagen, as well as the mutual relations of its basic types (I and III) in the extracellular matrix of the connective tissue. These changes are present not only in the area of hernial defects but at distant locations as well [2,3,4]. The authors of this report showed in 2011 [5] the differences in fluorescence spectra resulting from collagen destruction between biopsy samples harvested from the rectus sheath of hernia patients and healthy controls.

It is estimated that collagen represents 1/3rd of all body proteins, and 28 types of collagen have been identified from at least 46 distinctly differentiated chains. The structural unit of collagen, called the tropocollagen, is made up of three polypeptide chains bonded to each other by hydrogen bonds. Despite the super twisted nature of collagen, both N- and C-terminals are nonhelical telopeptide regions, and the tropocollagen helices bind to each other through appropriate cross-links and form well-defined spatial units [6]. Collagen, like any other protein, contains aromatic amino acids in its structure, but only some of them are capable of autofluorescence. Many types of collagen cross-links have been identified and described; however, very few of them exhibit the ability of autofluorescence. Therefore, fluorescence-emitting collagen amino acids and cross-links are the natural fluorescence markers in collagen, which can be probed experimentally in the case of isolated collagen, intracellular matrices, and all types of tissues.

The interest in the application of steady-state and time-resolved autofluorescence spectroscopy in biological sciences has significantly increased in recent years. Autofluorescence spectroscopy is a non-invasive, repeatable, spectrally selective, and highly sensitive method. Therefore, the use of these spectroscopic techniques in the life sciences seems to be very promising in the studies of different kinds of biological materials and their changes due to different diseases.

Reports in the literature on collagen fluorescence spectroscopy provide divergent information on the excitation and emission fluorescence spectra of collagen. In the Supplementary Material, we present examples of the literature on the fluorescence excitation and emission spectra of collagen, taken from references [7,8,9,10,11,12,13,14,15,16]. Those spectra differ in the various articles mentioned in the Supplementary Material, although each time they are referred to as the collagen fluorescence excitation and emission spectra. These differences are due to the fact that those spectra were obtained under different experimental conditions, i.e., at different fluorescence detection wavelengths (for the excitation spectra) and at different fluorescence excitation wavelengths (for the emission spectra). Given that collagen contains a large number of endogenous fluorophores differing in absorption bands and emission bands, it is obvious that different measuring conditions will yield different excitation and emission spectra of collagen fluorescence. This fact has to be taken into account when discussing the fluorescence properties of collagen and collagen-rich tissues.

The main aim of this article is to present a more systematic discussion of the steady-state and time-resolved autofluorescence spectroscopy of collagen isolated from bovine Achilles tendon. The excitation spectra of collagen fluorescence were measured for several detection wavelengths, and the emission spectra of collagen fluorescence were obtained for several excitation wavelengths. The spectra obtained were interpreted based on the measured excitation and emission fluorescence spectra of the three amino acids (i.e., phenylalanine, tyrosine, and tryptophan) and the fluorescence excitation and emission spectra for 13 collagen cross-links capable of autofluorescence, which have been thus far identified and reported in the literature. Measurements of collagen autofluorescence decays were performed for 5 excitation wavelengths, namely at 273 nm, 340 nm, 375 nm, 405 nm, and 450 nm. For each excitation wavelength, fluorescence decay was measured at several fluorescence emission wavelengths. The information obtained about the decay times of the measured fluorescence signals was discussed, taking into account the available (rather scarce) literature data recovered from similar studies on isolated collagen and collagen-rich tissues.

## 2. Results

### 2.1. Fluorescence Excitation and Emission Spectra of Collagen from Bovine Achilles Tendon

**Figure 1:**
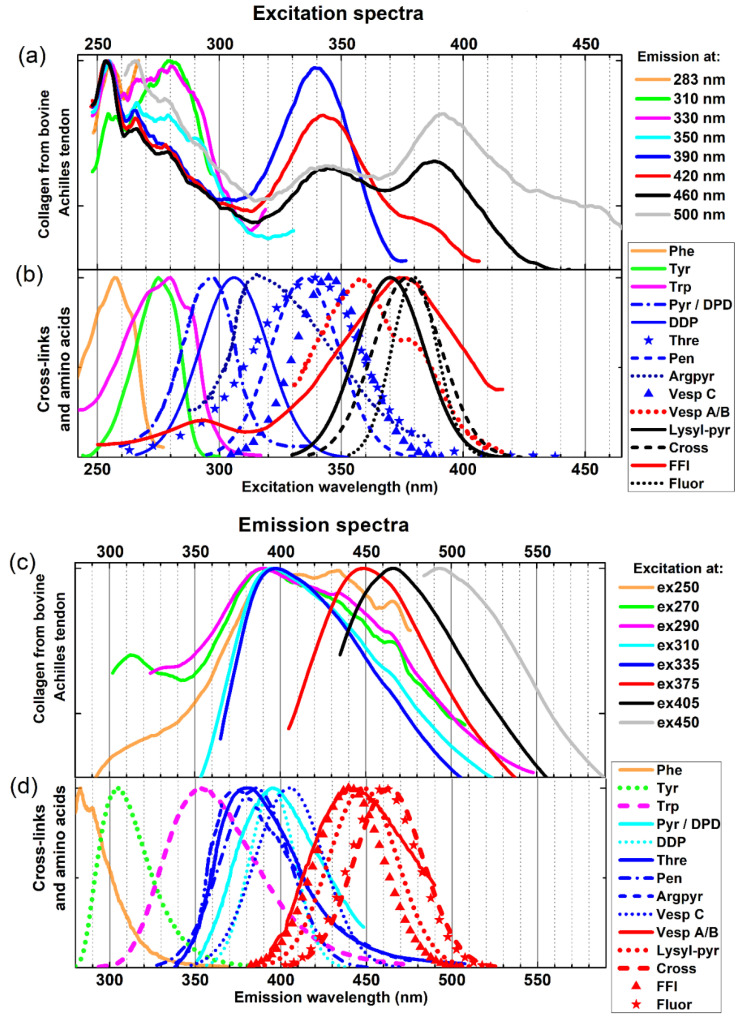
Fluorescence excitation (panel (**a**)) and emission (panel (**c**)) spectra of insoluble collagen powder from bovine Achilles tendon. Panels (**b**,**d**) display the excitation and emission fluorescence spectra of amino acids measured in water and similar simulated spectra of known fluorescent cross-links of collagen protein (see text for details).

In panels (a) and (c) of Figure 1 we show, correspondingly, the normalized-to-unity fluorescence excitation and emission spectra of collagen powder collected at different detection wavelengths of the emitted fluorescence (excitation spectra in Figure 1a) and at different excitation wavelengths (emission spectra in Figure 1c). The excitation and emission wavelengths used in the measurements are indicated in the legends on the right-hand sides of both panels.

The graphs of the excitation and emission spectra shown in Figure 1a,c indicate a greater number of fluorescent objects (chromophores capable of fluorescence) in collagen, which contribute to the detected fluorescence excitation and emission signals. The manifestation of these objects in the excitation spectra depends significantly on the fluorescence detection wavelength chosen, while in the emission spectra this manifestation strongly depends on the fluorescence excitation wavelength. The features of both types of obtained spectra become clearer after considering the plots of the excitation and emission spectra of the collagen constituent elements active in fluorescence spectroscopy, so far identified and discussed in the literature. Panel (b) of Figure 1 presents the plots of the excitation bands of 3 amino acids in aqueous solutions, namely phenylalanine (Phe), tyrosine (Tyr), and tryptophan (Trp), and also the known 13 collagen cross-links, isolated and identified by means of the HPLC methods, which are capable of fluorescence, namely pyridinoline (Pyr) [17,18], deoxypyridinoline (DPD) [18,19], 2,6-dimethyldifuro-8-pyrone (DDP) [19,20], threosidine (Thre) [21], pentosidine (Pen) [19,22,23], argpyrimidine (Argpyr) [23], vesperlysine C (Vesp C) [22,23], vesperlysine A and B (Vesp A/B) [22,23], lysyl-pyrropyridine (Lysyl-pyr) [23], crossline (Cross) [22,23], 2-(2-furoyl)-4(5)-(2-furanyl)-1H-imidazole (FFI) [23], and fluorolink (Fluor) [23]. The corresponding fluorescence emission bands for all the above-mentioned compounds are shown in panel (d) of Figure 1. The excitation spectra of amino acids Phe, Tyr, and Trp were collected experimentally at emission wavelengths $\lambda_{em}$ set at 283 nm, 305 nm, and 355 nm, while their emission bands were obtained at excitation wavelengths $\lambda_{ex}$ set to 255 nm, 273 nm, and 280 nm, respectively. In the case of the collagen cross-links Argpyr, FFI, Pyr, Thre, Ves A, and Ves B, the excitation and emission bands have been digitized from the article indicated in the third column of Table 1. For the rest of the cross-links, both kinds of spectra have been simulated by means of Gaussian function (just for visualization purposes) centered at the maxima, which was established experimentally and listed in Table 1 in the form of wavelength pairs $\lambda_{ex}/\lambda_{em}$. This was the only possibility to somehow illustrate these spectra, even in a very symbolic form, because the literature references indicated in the third column of Table 1 do not provide any information on the shapes of the excitation and emission bands for these cross-links.

In the excitation spectra of collagen from bovine Achilles tendon, presented in Figure 1a, three ranges of excitation wavelengths can be distinguished. In the 250–300 nm range, the collagen excitation spectra are correlated mainly with the 3 amino acids and FFI excitation spectra, as shown in panel (b) of Figure 1. The next 2 ranges that stand out are the 300–370 nm range and the 370–500 nm one. Collagen excitation spectra in both mentioned wavelength ranges can be correlated with the excitation spectra of two groups of collagen cross-links, i.e., with the group of cross-links with the excitation spectra shown in Figure 1b in blue (Pyr/DPD, DDP, Thre, Pen, Argpyr, and Vesp C), and with the group of cross-links whose excitation spectra are shown in the same Figure 1b in red and in black (Vesp A/B, Lysyl-pyr, Cross, FFI, and Fluor).

In the case of the emission spectra of the collagen from bovine Achilles tendon (Figure 1c), the situation is similar, i.e., three wavelength ranges in the fluorescence emission light can be distinguished, which are related to the three groups of chromophores discussed in the collagen excitation spectra. In the range of emission wavelengths 280–370 nm, the emitted collagen fluorescence is associated with the emission of the 3 amino acids, according to their fluorescence spectra shown in Figure 1d. In the emission wavelength range 370–430 nm, collagen fluorescence correlates with the fluorescence of the 1st group of cross-links distinguished in the case of excitation spectra, i.e., with the group of Thre, Pen, Argpyr, and Vesp C, while the collagen emission in the wavelength range 430–550 nm may be identified with the fluorescence of the 2nd group of cross-links, i.e., with the group of Vesp A/B, Lysyl-pyr, Cross, FFI, and Fluor.

On the subject of collagen spectral fluorescence spectroscopy, the literature (e.g., Refs. [7,8,9,10,11,12,13,14,15,16]) often provides very different information regarding the shapes and positions of the fluorescence excitation and emission bands of collagen (see Supplementary Material). This is because collagen contains a great number of different endogenous fluorophores, including aromatic amino acids, such as phenylalanine, tyrosine, and tryptophan, and fluorescent collagen cross-links. All of these fluorophores have different absorption spectra (some may overlap) and different fluorescence emission spectra (which may also overlap). Consequently, the fluorescence excitation spectra of collagen may be diametrically different depending on the detection wavelength of its fluorescence, and the fluorescence emission spectra of collagen may be substantially different depending on the excitation wavelength of its fluorescence. In other words, each time when presenting the measured fluorescence excitation and/or emission spectra of any type of collagen or any tissue containing various types of collagens, it is absolutely necessary to emphasize at which wavelengths λ*_ex_* and λ*_em_* these spectra were recorded. Changing λ*_ex_* or λ*_em_* will result in dramatically different fluorescence emission spectra or fluorescence excitation spectra.

It is noteworthy that the fluorescence spectrum of collagen excited at 450 nm (Figure 1c) may indicate that there are other spectroscopically active collagen cross-links that have not been yet identified (compare Figure 1c with Figure 1d). This observation requires further experimental exploration.

As clearly seen in the fluorescence excitation spectra of collagen shown in Figure 1a, the fluorescence detected at the wavelengths 390 nm, 420 nm, 460 nm, and 500 nm can be excited by the light in deep-UV. Furthermore, as seen in the collagen emission spectra shown in Figure 1c, for the excitations in the wavelength range of 250–310 nm, fluorescence at longer emission wavelengths (i.e., within 450–550 nm region) occurs and is emitted by the collagen cross-links alone. Both of these findings may mean that single- or multi-step excitation energy transfer processes from amino acids to the collagen cross-links (and further also between the cross-links themselves) are operative, assuming that the cross-links do not absorb the light in the 250–300 nm range or that this absorption is very low (see the excitation spectrum of FFI in Figure 1b). However, according to the excitation spectra shown in Figure 1b, the cross-links Pyr, DPD, DDP, and Argpyr can be the originally excited donor groups in the range of 250–300 nm, from which the excitation energy can be transferred to other collagen cross-links. Nevertheless, undoubtedly, this point needs further studies for elucidation, including time-resolved studies of preferably ultra-high temporal resolution.

### 2.2. Time-Resolved Autofluorescence Decays of Collagen from Bovine Achilles Tendon

**Figure 2:**
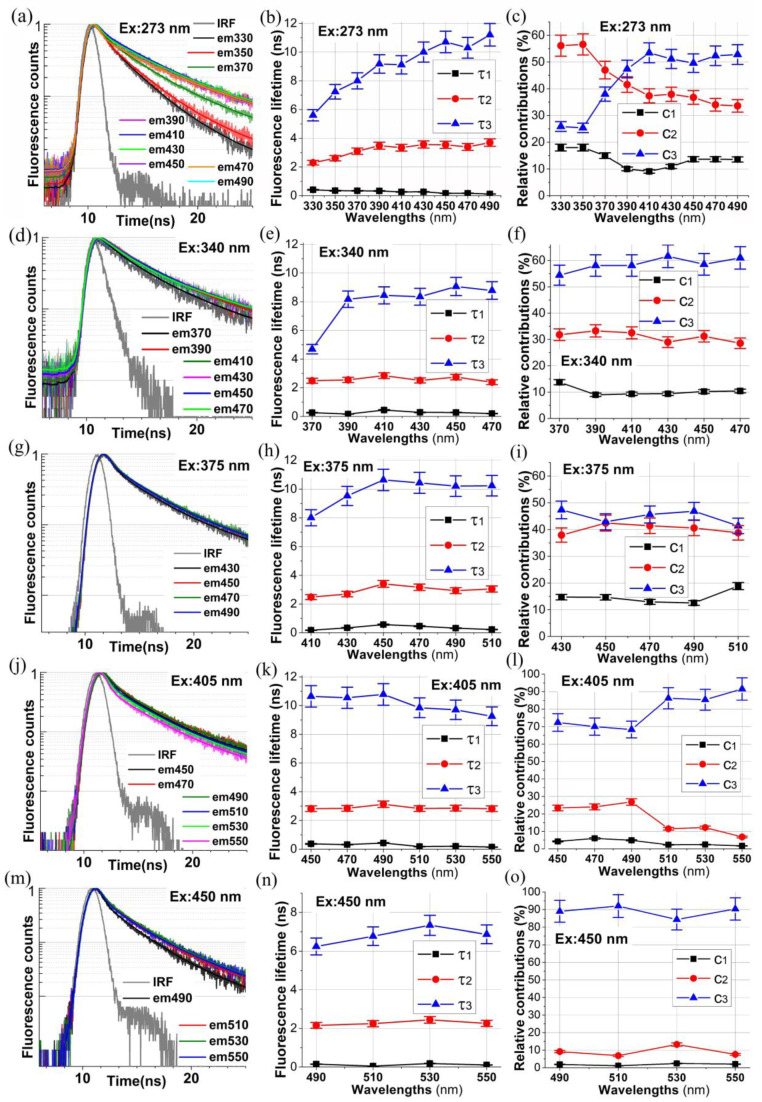
Autofluorescence decays of collagen (panels (**a**,**d**,**g**,**j**,**m**)), and the recovered decay parameters: decay times (panels (**b**,**e**,**h**,**k**,**n**)) and amplitudes (**c**,**f**,**i**,**l**,**o**) (see text).

The normalized histograms of fluorescence decays collected at 5 excitation wavelengths, namely at 273 nm, 340 nm, 375 nm 405 nm, and 450 nm, are shown in panels (a), (d), (g), (j), and (m) of Figure 2, correspondingly. For each of the exciting wavelengths, the histograms of autofluorescence decays (noisy data) were collected at different detection wavelengths, indicated in the legends of these panels. The solid curves represent the best fits of the three exponential fluorescence decays assumed as the model decay in the case of the samples studied, namely

(1) $$I\left(t\right)=\sum_{i=1}^{3}\alpha_{i}\exp\left(-t/\tau_{i}\right),$$

where $\tau_{i}$ and $\alpha_{i}$ (*i* = 1, 2, 3) are the three fluorescence decay times and the three amplitudes (contributions) of individual exponential decay to overall fluorescence decay I(t). The results of the numerical analysis of all histograms obtained are displayed in the rest of the panels of Figure 2. Panels (b), (e), (h), (k), and (n) show the estimated values of the sets of three fluorescence decay times $\left\{\tau_{i}\right\}$ (*i* = 1, 2, 3) for each analyzed histogram, while panels (c), (f), (i), (l), and (o) display the corresponding sets of three percentage contributions $\left\{c_{i}\right\}$ (*i* = 1, 2, 3) for each mono-exponential decay in I(t) being analyzed, and they are calculated from

(2) $$c_{i}=\alpha_{i}/(\alpha_{1}+\alpha_{2}+\alpha_{3})\times 100\%,$$

for each *i* (=1, 2, 3).

Table 2 summarizes the result of a numerical analysis of all collected autofluorescence decay histograms for the collagen powder from the bovine Achilles tendon studied. It must be emphasized here that, from the physical point of view, Equation (1) represents the time-evolution of fluorescence intensity I(t) due to excitation of the sample at a particular excitation wavelength λ*_ex_* and measured at particular emission wavelength λ*_em_* in the form of a linear combination of three mono-exponential basis functions $\exp(-t/\tau_{i})$, parameterized by three effective decay times $\left\{\tau_{i}\right\}\;$(*i*= 1, 2, 3), with the linear-combination parameters $\alpha_{i}$ (*i* = 1, 2, 3). The decay times $\tau_{i}$ depend on the excited-state lifetimes of all fluorophores probed in the experiment with the selected excitation λ*_ex_* and emission λ*_em_* wavelengths. The latter should be understood in such a way that depending on the selected λ*_ex_* and λ*_em_*, the spectral share (absorption and emission bands) of individual fluorophores changes, and thus the recovered values of $\left\{\tau_{i}\right\}$ (*i* = 1, 2, 3) may assume different values for different excitation and emission wavelengths.

As shown in Figure 2a, when exciting fluorescence at 273 nm, the fluorescence decays measured at the detection wavelengths of 330 nm and 350 nm decay over time much faster than the decays measured at the remaining detection wavelengths, i.e., in the range from 390 nm to 490 nm. For λ*_em_* = 330 nm and λ*_em_* = 350 nm, the fluorescence signal most likely comes from tyrosine and tryptophan, which can be inferred from the values of the dominant fluorescence decay time $\tau_{2}$, which are at the level of about 2 ns–3 ns. Indeed, according to the literature data, the kinetic decay of tyrosine fluorescence, when excited with a wavelength λ*_ex_* = 287 nm and when detecting fluorescence at a wavelength of λ*_em_* = 303 nm, is bi-exponential, with decay times at the level of $\tau_{1}$ = 0.36 ns and $\tau_{2}$ = 3.1 ns [24]. However, in the case of tryptophan, for λ*_ex_* = 296 nm and λ*_em_* = 350 nm, the kinetic decay of fluorescence in ethanol is bi-exponential, with the decay times of $\tau_{1}$ = 0.152 ns and $\tau_{2}$ = 1.78 ns [25]. For tryptophan in proteins, under the same experimental conditions, the 2nd fluorescence decay time $\tau_{2}$ changes from 1.25 ns to 3.57 ns [25]. Thus, our conclusion as to the origins of fluorescence decays detected at λ*_em_* = 330 nm and λ*_em_* = 350 nm seems to be justified.

For λ*_em_* = 370 nm, the decay of the fluorescence begins to show the contribution of the collagen cross-links fluorescence. As can be seen in panel (c) of Figure 2, at λ*_em_* = 380 nm, the contributions of fluorescence decay components with the decay times $\tau_{2}$ (at the level of about 3.5 ns) and $\tau_{3}$ (at the level of about 9 ns) are comparable. The value of $\tau_{3}$ at the level of about 9 ns corresponds well to the collagen cross-links fluorescence decay time. In fact, the decay of fluorescence for Type II collagen powder, when excited with λ*_ex_* = 337 nm and when detecting fluorescence at λ*_em_* = 400 nm, is bi-exponential, with a longer decay time $\tau_{2}$ = 6.7 ns [26]. In addition, for Type I insoluble Achilles tendon collagen, after excitation at the same wavelength and detection at 390 nm, the fluorescence decay is also bi-exponential, with decay times of $\tau_{1}$ = 3.9 ns and $\tau_{2\;}$= 9.9 ns for dry collagen and with decay times of $\tau_{1}$ = 2.7 ns and $\tau_{2}$ = 8.4 ns for hydrated collagen [27].

The discussion of fluorescence decays excited by light with wavelengths of 340 nm, 375 nm, and 405 nm is less complicated, because for all detection wavelengths of fluorescence light collected, the values of decay time $\tau_{3}$ (at the level of about 8 ns–11 ns) are characteristic for the decay time of collagen cross-link fluorescence [26,27]. This component of overall fluorescence decays *I* (*t*) definitely dominates according to Figure 2o.

The case of collagen fluorescence excited at the wavelength of 450 nm is very intriguing. Additionally, in this case, the component of fluorescence decay with decay time $\tau_{3}$ dominates the time evolution of I(t). However, this time the values of $\tau_{3}$ in the fluorescence detection wavelength range from 490 nm to 550 nm take the values in between of about 6 ns and about 7 ns. These values significantly differ from the ones of $\tau_{3}$ obtained for the previously discussed 3 fluorescence excitation wavelengths of 340 nm, 375 nm, and 405 nm. This finding further supports our conclusion drawn in Section 2.1 concerning the probable existence of thus-far unidentified collagen cross-links, which can be excited at this excitation wavelength.

In other words, our results of steady-state and time-resolved fluorescence studies of collagen from bovine Achilles tendon indicate that it is very likely that there exist additional collagen cross-links, which can be excited at the wavelength of 450 nm, and which have not been yet isolated and identified. Undoubtedly, this hypothesis needs further exploration.

In the previous section, when discussing the fluorescence excitation spectra of the collagen powder detected at the emission wavelengths of 390 nm, 420 nm, 460 nm, and 500 nm (Figure 1a,b), and also the recorded emission bands excited in the deep-UV region at 250 nm, 270 nm, 290 nm, and 310 nm (Figure 1c,d), we concluded that the fluorescence excitation energy of amino acids can be non-radiatively transferred to collagen cross-links, i.e., the amino acids are being excited in the deep-UV and the emission from the collagen cross-links occurs (in the region of 390 nm–550 nm). A similar conclusion can be drawn from the time-resolved data. Indeed, with the excitation at 273 nm (Figure 2a–c), the fluorescence decays collected at 330 nm and 350 nm originate from the amino acids tyrosine and tryptophan. However, for the detection wavelengths from 390 nm to 490 nm, the measured decays evidently come from the fluorescence decays of the collagen cross-links. The excited-state evolution of collagen cross-links directly at 340 nm (Figure 2d–f), at 375 nm (Figure 2g–i), at 405 nm (Figure 2j–l), and at 450 nm (Figure 2m–o) supports our conclusion.

When the fluorescence excitation energy transfer occurs, one of the pre-exponential factors in the bi- or multi-exponential time-evolution of the energy acceptor fluorescence signal should take a negative value. Such an exponential component in overall fluorescence intensity means the increase in fluorescence signal due to the pumping of the excited-state population of energy acceptors via the excitation energy transfer. However, according to Table 2, none of the pre-exponential factors (amplitudes) corresponding to the shorter decay times $\tau_{1}$ and $\tau_{2}$, in the case of fluorescence excitation at 273 nm and detection at 390–490 nm, take a negative value. We must remember, however, that the excitation pulse width in our experiment was about 900 ps, and the channel width was set to 24 ps. Therefore, most likely, the time resolution of our experiment was too low to detect the component with the rise time in the fluorescence signal detected. Most likely, the excitation energy process from amino acids to collagen cross-links or between the collagen cross-links themselves proceeds on a much shorter time scale compared to the time resolution of our experiment. Thus, additional elucidating time-resolved fluorescence studies of collagen with excitations in the deep-UV region of appropriately high time resolution (of femtosecond or single picosecond resolution) are definitely required to further explore this point.

## 3. Discussion

The obtained spectral results demonstrate substantial changes in the fluorescence excitation and emission bands of collagen powder isolated from bovine Achilles tendon depending on the excitation and detection wavelengths chosen in the experiment. In other words, each time when presenting the measured fluorescence excitation and/or emission spectra of any type of collagen or any tissue containing various types of collagen, it is absolutely necessary to emphasize at which wavelengths λ*_ex_* and λ*_em_* these spectra were recorded. Changing λ*_ex_* or λ*_em_* will result in recording markedly different fluorescence emission spectra or fluorescence excitation spectra. Furthermore, the excitation and emission bands of collagen recorded in our study provide a clear-cut indication that it is very likely that there exist additional collagen cross-links not identified so far, which can be excited at longer excitation wavelengths (450 nm in our study). Therefore, further research to identify additional collagen cross-links is highly recommended.

In addition, the excitation spectra measured at longer emission wavelengths at which the collagen cross-links emit the fluorescence light and the emission spectra obtained for the excitation in deep-UV region, as well as the results of time-resolved fluorescence studies with the excitation in deep-UV region and detection at longer emission wavelengths (390 nm–550 nm), suggest that it is very likely that the fluorescence excitation energy transfers from amino acids to collagen cross-links and also between the cross-links themselves. This transfer, if present, very likely occurs on a very short time scale, i.e., it is an ultra-fast phenomenon on a femtosecond or single picosecond time scale. This point needs further experimental exploration with the application of appropriately fast time-resolved fluorescence techniques.

Finally, to better understand the nature of collagen fluorescence of all thus-far isolated and identified (and possibly the new) cross-links should be subjected to very systematic spectroscopic investigations, including stationary ones (the excitation and emission spectra) and also time-resolved ones, enabling to recover the fluorescence decay times of all individual collagen cross-links. Obtaining more detailed information on the stationary and time-resolved spectroscopic properties of collagen and its endogenous fluorophores can be very important in the interpretation and better understanding of the results of fluorescence studies of collagen and collagen-rich tissues in a wide area of biomedicine.

## 4. Materials and Methods

Phenylalanine, tyrosine, tryptophan, and collagen powder from bovine Achilles tendon (C9879) were purchased from Sigma-Aldrich and were used as received. Collagen powder samples were placed between two quartz plates. The fluorescence excitation and emission bands were acquired using Horiba Jobin Yvon Fluoromax 4 spectrofluorometer (HORIBA Jobin Yvon S.A.S., 16-18 rue du Canal, 91165 Longjumeau, France), at the “front-face” experimental geometry. Particular attention was paid to arranging the samples in such a way that the light-beam exciting fluorescence did not hit the detector. The fluorescence excitation spectra were collected at fluorescence detection wavelengths of 283 nm, 310 nm, 330 nm, 350 nm, 390 nm, 420 nm, 460 nm, and 500 nm, while the fluorescence emission bands were measured at excitation wavelengths of 250 nm, 270 nm, 290 nm, 310 nm, 335 nm, 375 nm, 405 nm, and 450 nm. In both cases, the spectra were scanned at the speed of 300 nm/min with 3 repetitions, and the scanning range was set to 200–600 nm. The widths of emission and excitation slits were set to 5 nm. The obtained excitation and emission spectra were analyzed using Origin 9.1 software, v. 9.1 (OriginLab Corp.,Northampton, MA, USA). Time-resolved fluorescence studies were performed using an FI900cd fluorometer (Edinburgh Instruments, Edinburgh, Scotland), employing 5 diodes emitting pulsed light at 5 different wavelengths, namely 273 nm, 340 nm, 375 nm, 405 nm, and 450 nm. The half-width of the generated pulses was 900 ps, and the spectral half-width for these diodes was approximately 30 nm. The excitation light passed through the monochromator, thanks to which the spectral width of the excitation pulses was fixed at 10 nm. Similarly, in the detection channel, the fluorescence light passed through the monochromator, selecting a predetermined wavelength of the emitted signal with a spectral width of 10 nm. The intensity of the fluorescence excitation light was controlled with a gray filter. Likewise, in the detection channel, the incoming fluorescence intensity to the detection channel was also regulated by a gray filter. Histograms of fluorescence decays were acquired (in 1024 channels; channel width 24 ps) for different detection wavelengths ranging from 330 nm to 580 nm; they were chosen depending on the wavelength of exciting light. The repetition rate of the diodes was set to 10 MHz. The instrument response function (IRF) on the exciting pulse of light was collected as the scattered light by nanoparticles suspension (Ludox, Sigma-Aldrich, St. Louis, Missouri, USA). Obtained histograms were analyzed using F900 software, v. 7.2.2 (Edinburgh Instruments, Edinburgh, Scotland), and graphical presentation of obtained results was performed with Origin 9.1 software.

## Figures and Tables

**Table 1:** The isolated and identified fluorescence-capable cross-links of collagen, together with the wavelengths corresponding to the maxima of fluorescence excitation and emission spectra.

Cross-Link	Ex/Em [nm]	References
Trivalent cross-link		
Pyridinoline (Pyr),	295 (acid), 325 (neutral)/340 (alkaline)/395	[17,18]
Deoxypyridinoline (DPD)	294 (acid), 325 (neutral)/395	[18,19]
2,6-dimethyldifuro-8-pyrone (DDP)	306/395	[19,20]
Advanced glycation endproducts (AGEs)		
Threosidine (Thre)	328/402	[21]
Pentosidine (Pen)	335/385	[19,22,23]
Argpyrimidine (Argpyr)	335/400	[23]
Vesperlysine C (Vesp C)	345/405	[22,23]
Vesperlysine A/B (Vesp A/B)	366/442	[22,23]
Lysyl-pyrropyridine (Lysyl-pyr)	370/448	[23]
Crossline (Cross)	379/463	[22,23]
2-(2-furoyl)-4(5)-(2-furanyl)-1H-imidazole (FFI)	380/440	[23]
Fluorolink (Fluor)	380/460	[23]

**Table 2:** Fluorescence decay times $\left\{\tau_{i}\right\}$ (*i* = 1, 2, 3) and the corresponding percentage contributions $\left\{c_{i}\right\}$ (*i* = 1, 2, 3) obtained for all autofluorescence decays analyzed (see text).

λ*_ex_* [nm]	λ*_em_* [nm]	τ_1_/*c*_1_ [ns]/[%]	τ_2_/*c*_2_ [ns]/[%]	τ_3_/*c*_3_ [ns]/[%]
273	330	0.410/18	2.30/56	5.60/26
	350	0.348/18	2.61/57	7.24/25
	370	0.340/15	3.10/47	8.00/38
	390	0.329/10	3.48/42	9.17/48
	410	0.265/9	3.34/37	9.12/54
	430	0.269/11	3.57/38	10.00/51
	450	0.168/14	3.54/37	10.71/49
	470	0.169/14	3.40/34	10.30/52
	490	0.120/13	3.70/34	11.20/53
340	370	0.264/14	2.49/32	4.69/54
	390	0.167/9	2.54/33	8.17/58
	410	0.442/9	2.84/33	8.44/58
	430	0.285/9	2.51/29	8.35/62
	450	0.277/10	2.74/31	9.06/59
	470	0.201/10	2.38/29	8.78/61
375	430	0.339/15	2.70/38	9.53/47
	450	0.565/15	3.40/42	10.64/43
	470	0.459/13	3.17/41	10.44/46
	490	0.319/12	2.93/41	10.21/47
	510	0.229/19	3.05/39	10.24/42
405	450	0.364/5	2.82/23	10.64/72
	470	0.319/6	2.84/24	10.54/70
	490	0.427/5	3.13/27	10.78/68
	510	0.182/3	2.82/11	9.85/86
	530	0.203/2	2.86/12	9.71/85
	550	0.148/2	2.82/7	9.25/91
450	490	0.148/2	2.16/9	6.24/89
	510	0.049/2	2.24/7	6.77/91
	530	0.180/3	2.44/13	7.33/84
	550	0.105/3	2.26/7	6.87/90

## Data Availability

The data presented in this study are available in the article, and raw data are available upon request.

## Supplementary Materials

The supplementary material can be downloaded at: https://www.mdpi.com/article/10.3390/ijms24087631/s1
