# Supplementary material for: On the Nature of Stationary and Time-Resolved Fluorescence Spectroscopy of Collagen Powder from Bovine Achilles Tendon
Source: Int J Mol Sci. 2023 Apr 21;24(8):7631. doi: 10.3390/ijms24087631 (PMC10145534; doi:10.3390/ijms24087631)
Supplement: Supplementary file 1 [file ijms-24-07631-s001.zip › ijms-2303304-supplementary.pdf]

Supplementary Material

Figure S1 displays the differences in fluorescence emission bands of collagen prepared by different methods. The spectra have been taken (digitized) from the following literature references:

1. Insoluble collagen from human diabetic/nondiabetic skin solubilized by collagenase (excitation at 370 nm).

   Monnier, V.M.; Kohn, R.R.; Cerami, A. Accelerated age-related browning of human collagen in diabetes mellitus. *Proc. Natl. Acad. Sci. USA* **1984**, *81*, 583–587. https://doi.org/10.1073/pnas.81.2.583.

2. Insoluble collagen from rat diabetic/nondiabetic skin solubilized by collagenase (excitation at 370 nm).

   Monnier, V.M.; Kohn, R.R.; Cerami, A. Accelerated age-related browning of human collagen in diabetes mellitus. *Proc. Natl. Acad. Sci. USA* **1984**, *81*, 583–587. https://doi.org/10.1073/pnas.81.2.583.

3. Insoluble collagen from rat skin solubilized by collagenase (excitation at 370 nm).

   Suárez, G.; Rajaram, R.; Bhuyan, K.C.; Oronsky, A.L.; Goidl, J.A. Administration of an aldose reductase inhibitor induces a decrease of collagen fluorescence in diabetic rats. *J. Clin. Invest.* **1988**, *82*, 624–627. https://doi.org/10.1172/JCI113641.

4. Acid-soluble collagen from tail tendons of young albino rats (excitation at 275 nm).

   Sionkowska, A.; Kamińska, A. Changes induced by ultraviolet light in fuorescence of collagen in the presence of b-carotene. J. Photochem. Photobiol. *A Chem.* **1999**, *120*, 207–210. https://doi.org/10.1016/S1010-6030(98)00427-4.

5. Cosmetic collagen IV dispersed in water (excitation at 370 nm).

Smirnova, O.D.; Rogatkin, D.A.; Litvinova, K.S. Collagen as in vivo quantitative fluorescent biomarkers of abnormal tissue changes. *J. Innov. Opt. Health Sci.* **2012**, *5*, 1250010. https://doi.org/10.1142/S1793545812500101.

6. Acid- and pepsin-soluble collagen from bovine hide (excitation at 253 nm).

   Wu, K.; Liu, W.; Li, G. The aggregation behavior of native collagen in dilute solution studied by intrinsic fluorescence and external probing. Spectrochim. *Acta A Mol. Biomol. Spectrosc.* **2013**, *102*, 186–193. https://doi.org/10.1016/j.saa.2012.10.048.

7. Hydrolyzed collagen powder (excitation at 360 nm).

   Ionita, I.; Dragnea, A.M.; Gaidaub, C.; Dragomirb, T. Collagen fluorescence measurements on nanosilver treated leather. *Rom. Rep. Phys.* **2010**, *62*, 634–643.

8. Acid-soluble collagen from calf skin (excitation at 345 nm).

   Deyl, Z.; Praus, R.; Šulcová, H.; Goldman, J.N. Fluorescence of collagen—Properties of tyrosine residues and another fluorescent element in calf skin collagen. *FEBS Lett.* **1969**, *5*, 187–191. https://doi.org/10.1016/0014-5793(69)80328-5.

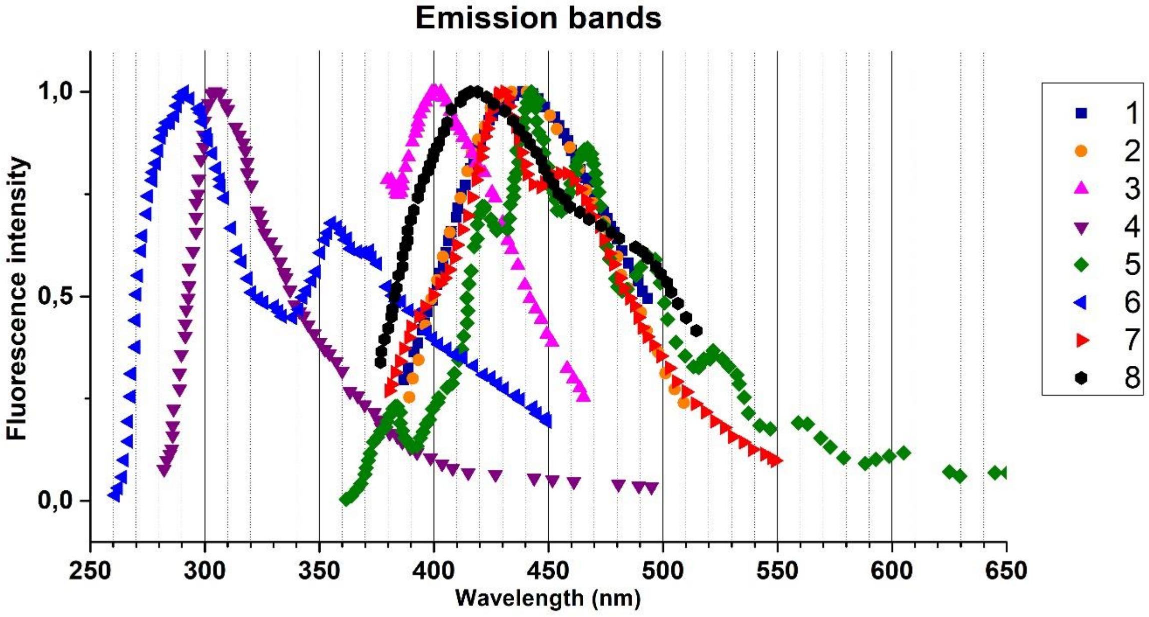

Figure S1. Examples of emission spectra referred to in the literature as fluorescence emission bands of collagen.

Figure S2 shows the differences in fluorescence excitation spectra of collagen prepared by distinct methods. The spectra have been taken (digitized) from the following literature references:

1. Insoluble collagen from human diabetic/nondiabetic skin solubilized by collagenase (emission at 440 nm).

   Monnier, V.M.; Kohn, R.R.; Cerami, A. Accelerated age-related browning of human collagen in diabetes mellitus. *Proc. Natl. Acad. Sci. USA* **1984**, *81*, 583–587. https://doi.org/10.1073/pnas.81.2.583.

2. Insoluble collagen from rat diabetic/nondiabetic skin solubilized by collagenase (emission at 440 nm).

Monnier, V.M.; Kohn, R.R.; Cerami, A. Accelerated age-related browning of human collagen in diabetes mellitus. *Proc. Natl. Acad. Sci. USA* **1984**, *81*, 583–587. https://doi.org/10.1073/pnas.81.2.583.

3. Pepsin-digestible collagen from mice skin (emission at 400 nm).

   Kollias, N.; Gillies, R.; Moran, M.; Kochevar, I.E.; Anderson, R.R. Endogenous Skin Fluorescence Includes Bands that may Serve as Quantitative Markers of Aging and Photoaging. *J. Invest. Dermatol.* **1998**, *111*, 776–780. https://doi.org/10.1046/j.1523-1747.1998.00377.x.

4. Collagenase-digestible collagen from mice skin (emission at 480 nm).

   Kollias, N.; Gillies, R.; Moran, M.; Kochevar, I.E.; Anderson, R.R. Endogenous Skin Fluorescence Includes Bands that may Serve as Quantitative Markers of Aging and Photoaging. *J. Invest. Dermatol.* **1998**, *111*, 776–780. https://doi.org/10.1046/j.1523-1747.1998.00377.x.

5. Excitation band of human skin compared with pepsin-digestible collagen (emission at 390 nm).

   Gillies, R.; Zonios, G.; Anderson, R.R.; Kollias, N. Fluorescence Excitation Spectroskopy Provides Information About Human Skin In Vivo. *J. Invest. Dermatol.* **2000**, *115*, 704–707. https://doi.org/10.1046/j.1523-1747.2000.00091.x.

6. Excitation band of human skin compared with collagenase-digestible collagen (emission at 460 nm).

   Gillies, R.; Zonios, G.; Anderson, R.R.; Kollias, N. Fluorescence Excitation Spectroskopy Provides Information About Human Skin In Vivo. *J. Invest. Dermatol.* **2000**, *115*, 704–707. https://doi.org/10.1046/j.1523-1747.2000.00091.x.

7. Acid-soluble collagen from calf skin (emission at 440 nm).

Deyl, Z.; Praus, R.; Šulcová, H.; Goldman, J.N. Fluorescence of collagen—Properties of tyrosine residues and another fluorescent element in calf skin collagen. *FEBS Lett.* **1969**, *5*, 187–191. https://doi.org/10.1016/0014-5793(69)80328-5.

8. Collagen bought from Mallinckrodt Baker, INC (Stokes shift spectra with max excitation at 339,5 nm).

Pu, Y.; Wang, W.; Yang, Y.; Alfano, R.R. Stokes shift spectroscopic analysis of multifluorophores for human cancer detection in breast and prostate tissues. *J. Biomed. Opt.* **2013**, *18*, 17005. https://doi.org/10.1117/1.JBO.18.1.017005.

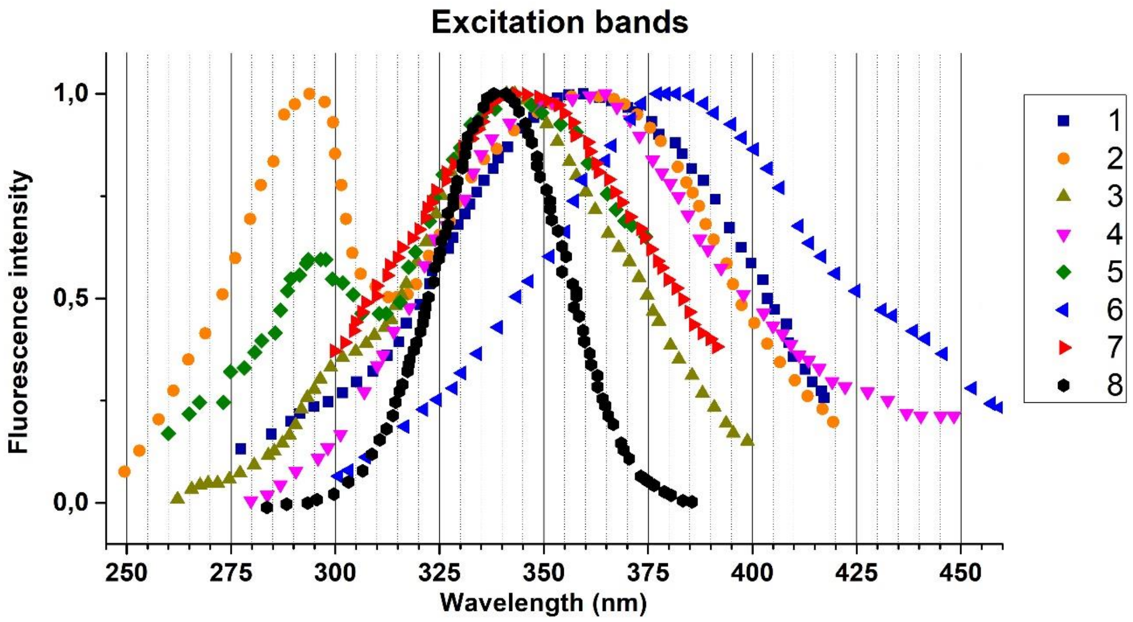

Figure S2 Examples of excitation spectra referred to in the literature as fluorescence excitation bands of collagen.
